# Quantitative analysis of ocular deviation under eye occlusion: a descriptive study using the ORTe EYENAC eye-tracking system

**DOI:** 10.1186/s12886-025-04101-z

**Published:** 2025-05-06

**Authors:** Shunya Tatara, Yuna Magara, Touko Sasaki, Fumiatsu Maeda, Noriaki Murata, Kazuhiro Itagaki, Tomoya Handa, Haruo Toda

**Affiliations:** 1 Department of Orthoptics and Visual Sciences, Niigata University of Health and Welfare, Niigata, Japan; 2 Major in Health and Welfare, Niigata University Graduate School of Health and Welfare, Niigata, Japan; 3 Nagaoka Eye Clinic, Nagaoka, Japan; 4 Department of Rehabilitation, Orthoptics and Visual Science Course, School of Allied Health Sciences, Kitasato University, Sagamihara, Japan; 5 Present Address: Department of Ophthalmology, Faculty of Medicine and Graduate School of Medicine, Hokkaido University, Sapporo, Japan; 6 Present Address: Department of Ophthalmology, Kanazawa Medical University, Kahoku, Japan

**Keywords:** Ocular deviation, Exophoria, Eye-tracking, ORTe EYENAC, Video-oculography, Deviation stabilization, Deviation speed, Cover test

## Abstract

**Background:**

The cover–uncover test is traditionally used in the qualitative assessment of heterotropia and heterophoria, while the alternate prism cover test (APCT) quantifies ocular deviations. However, the APCT is time-consuming and prone to interexaminer variability. Video-oculography technology offers a promising alternative for quantifying ocular deviation under occlusion. This study aims to visualize ocular deviation during occlusion and determine the time it takes for deviation to stabilize in participants with exodeviation using the ORTe EYENAC eye-tracking system.

**Methods:**

The study participants included 15 university students aged 20–22 years, among whom 13 had exophoria, 1 had intermittent exotropia, and 1 had exotropia. Eye position was measured using the ORTe EYENAC, which records gaze when one eye is occluded. Data were fitted to a logistic function to estimate ocular deviations, deviation speed, and stabilization time. The overshoot depth was defined as the maximum deviation beyond the final position before stabilization. Spearman’s rank correlation coefficient analyzed the correlations between the deviation angle, speed, and stabilization time.

**Results:**

The fusion-free eye position stabilized at 3.33 ± 2.39 s among those with exophoria. Significant correlations were found between the deviation angle and deviation speed (r_s_ = − 0.582, *p* = 0.0403), as well as between the deviation angle and stabilization time (r_s_ = 0.663, *p* = 0.0135). An overshoot of > 1°, 0.5°–1.0°, and < 0.5° was seen in 3, 4, and 6 patients, respectively. The overshoot depth also correlated with deviation speed (r_s_ = 0.775, *p* = 0.0029).

**Conclusions:**

Ocular deviation under occlusion was effectively visualized in participants with exodeviation using the ORTe EYENAC, providing a clear representation of eye movement during the cover test. Among participants with exophoria, the fusion-free eye position stabilized at an average of 3.33 ± 2.39 s. However, the stabilization time varied with the angle of ocular deviation, suggesting that the occlusion time needs to be individually designed based on the angle of strabismus.

## Background

The cover–uncover test (CUT) is a method for qualitatively assessing heterotropia and heterophoria. Heterotropia is detected by examining the movement of the other eye when one eye is covered, while heterophoria is detected by observing the movement of the eye when uncovered. Ocular deviation is often quantified using the alternate prism cover test (APCT) and single prism cover test (SPCT). The APCT can quantify both latent and manifest aspects of ocular deviation [1], whereas the SPCT can only quantify manifest deviation. The prism cover test requires the examination to be performed while changing the prism power multiple times. If both vertical and horizontal deviations are present, then the examination must be continued while changing the vertical prism power multiple times after determining the horizontal deviation. As a result, the quantitative values for APCT can vary depending on the examiner [2, 3]. However, if the amount of deviation under occlusion during CUT is known, then the quantitative evaluation of heterotropia and heterophoria can be simultaneously performed alongside qualitative testing.

Video-oculography (VOG) technology has gained increasing attention in recent years and is being applied in eye position testing [4–9]. By observing the gaze movement of the binocular eye position and fusion-free eye position, heterotopia and heterophoria can be qualitatively evaluated. Additionally, by calculating the angle of the gaze, ocular deviation can be quantitatively evaluated. Manifest ocular deviation can be quantified using the angle of the binocular eye position, whereas both latent and manifest deviation can be quantified using the angle of the fusion-free eye position. In the cover test, fusion is eliminated by occluding one eye, and sufficient ocular deviation can be detected. However, while measuring the fusion-free eye position, there is no standard time for each eye to be occluded. Holmes et al. [10] suggested that APCT might be confounded by differing periods of dissociation, revealing more or less of an underlying phoric component. Such variability in technique might lead to greater variability in the deviations measured by APCT. Since the cover test method cannot observe eye deviation under occlusion, the time it takes for the eye deviation to stabilize after occlusion remains unknown.

The ORTe EYENAC [11] is a device that can analyze eye gaze even when one eye is occluded, made possible through the combination of a visual stimulus display, a hot mirror, and two eye cameras dedicated for each eye. This device makes it possible to observe eye deviation under occlusion. This study aimed to visualize eye deviation under occlusion and to determine the time it takes for eye deviation to stabilize during the eye position examination using the ORTe EYENAC eye-tracking system in participants with exodeviation.

## Methods

### Participants and measurements

The study participants included 15 university student volunteers (20–22 years old) who were examined by an ophthalmologist and had no eye diseases other than eye position and refractive error. The participants’ eye position was confirmed by a conventional cover test before the experiment: 13 participants had exophoria, 1 had intermittent exotropia, and 1 had exotropia. All participants underwent quantification of ocular deviation using the APCT. To ensure consistency with the testing distance of the ORTe EYENAC, the APCT was performed using a near target. The data were anonymized prior to analysis.

### Measurement device

**Fig. 1:**
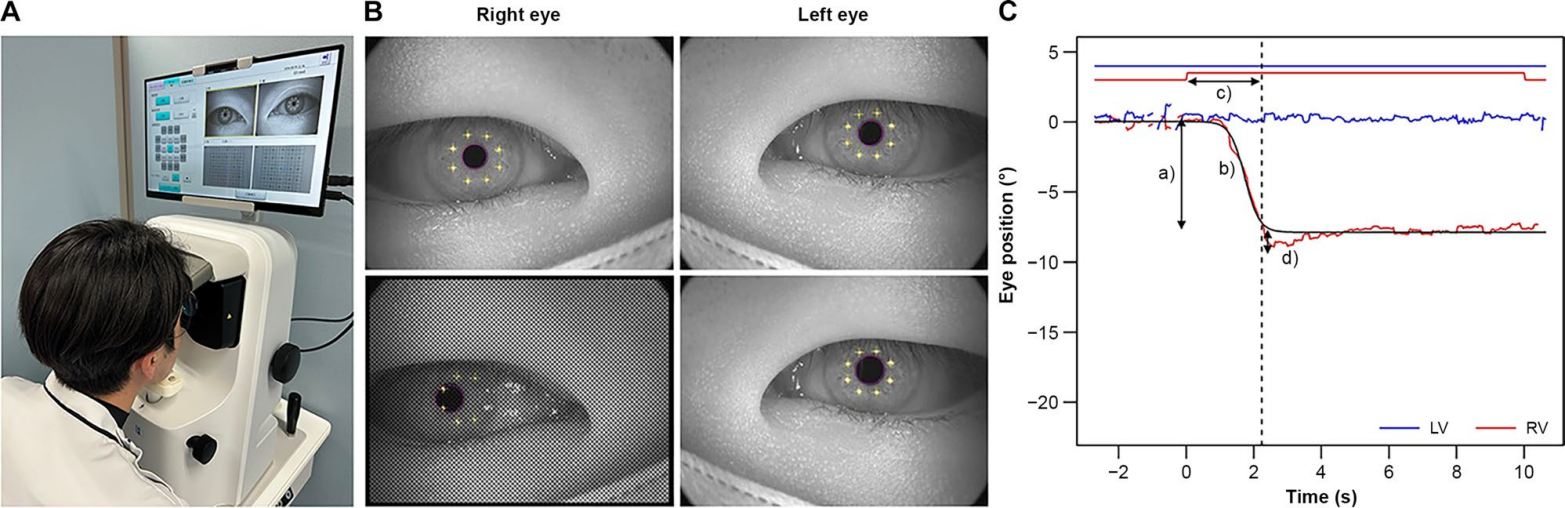
Measurement method **A**. The state of the ocular deviation measurement by ORTe EYENAC. **B**. The gaze is detected from the corneal reflex and the center of the pupil. Upper image: eye is not occluded; lower image: right eye is occluded. **C**. Sample results of exophoria. The start of occlusion was set as 0 s, with the time before and after occlusion represented with negative and positive values, respectively. The straight line at the top of the figure indicates nonocclusion, and the protrusion indicates occlusion. The dotted line indicates the time when the deviation stabilized. Each waveform shows the change in eye position corresponding to the y-axis, and a logistic curve has been fitted to the deviation of the right eye

The ORTe EYENAC (JAPAN FOCUS COMPANY, LTD., Tokyo, Japan) was used for the measurements (Fig. 1A). This device is equipped with an internal liquid crystal display (LCD) monitor for each eye, and fusion-free eye position was measured by turning off the monitor of one eye. The size of the internal LCD monitor was 6 inches, with a resolution of 2560 × 1440 pixels. An independent eye camera records each eye, and the gaze can be recorded regardless of whether the monitor is on or off. The dot on the LCD monitor was designed to be viewed through the eyepiece at 33 cm [12]. Before starting the eye position measurement, calibration was performed following the device’s standard procedure [11]. Calibration involved monocular fixation on a single dot displayed directly in front of each eye. All the participants completed the calibration successfully within a few seconds. The amount of ocular deviation was defined as the difference between the gaze of the fixation eye and the contralateral eye with the monitor turned off and the fixation target hidden (Fig. 1B). The sampling rate for recording the eye position was 30 Hz.

### Analysis

The eye position data 10 s before and after occlusion measured with ORTe EYENAC was output and fitted with a logistic function (the black line in Fig. 1C). Ocular deviations were obtained based on the difference between the two asymptotes of the function (denoted as *a* in Fig. 1C). The peak eye velocities were estimated using the maximum slope of the function (denoted as *b* in Fig. 1C). The time required for ocular deviation was determined as the time to reach 90% of the obtained ocular deviation (the dotted line in Fig. 1C). In some cases, the eye position once went beyond the final position before reaching it. To evaluate these temporary “overshoot” movements, the maximum ocular deviation was also measured for every trial (denoted as *d* in Fig. 1C).

For participants with exophoria, we evaluated the correlations between the angle of deviation and deviation speed, between the angle of deviation and time until the fusion-free eye position was stabilized, and between the overshoot depth and deviation speed. Spearman’s rank correlation coefficient was used to calculate the correlation coefficient.

Fitting eye positional data to a logistic function and statistical analyses were performed on R (https://www.r-project.org) for Mac (Mac mini 2018, Apple computer).

### Ethics approval and consent to participate

This study was approved by the Niigata University of Health and Welfare committee (19274–240513). The experiment was conducted in accordance with the Declaration of Helsinki, and written informed consent was obtained from all participants.

## Results

**Fig. 2:**
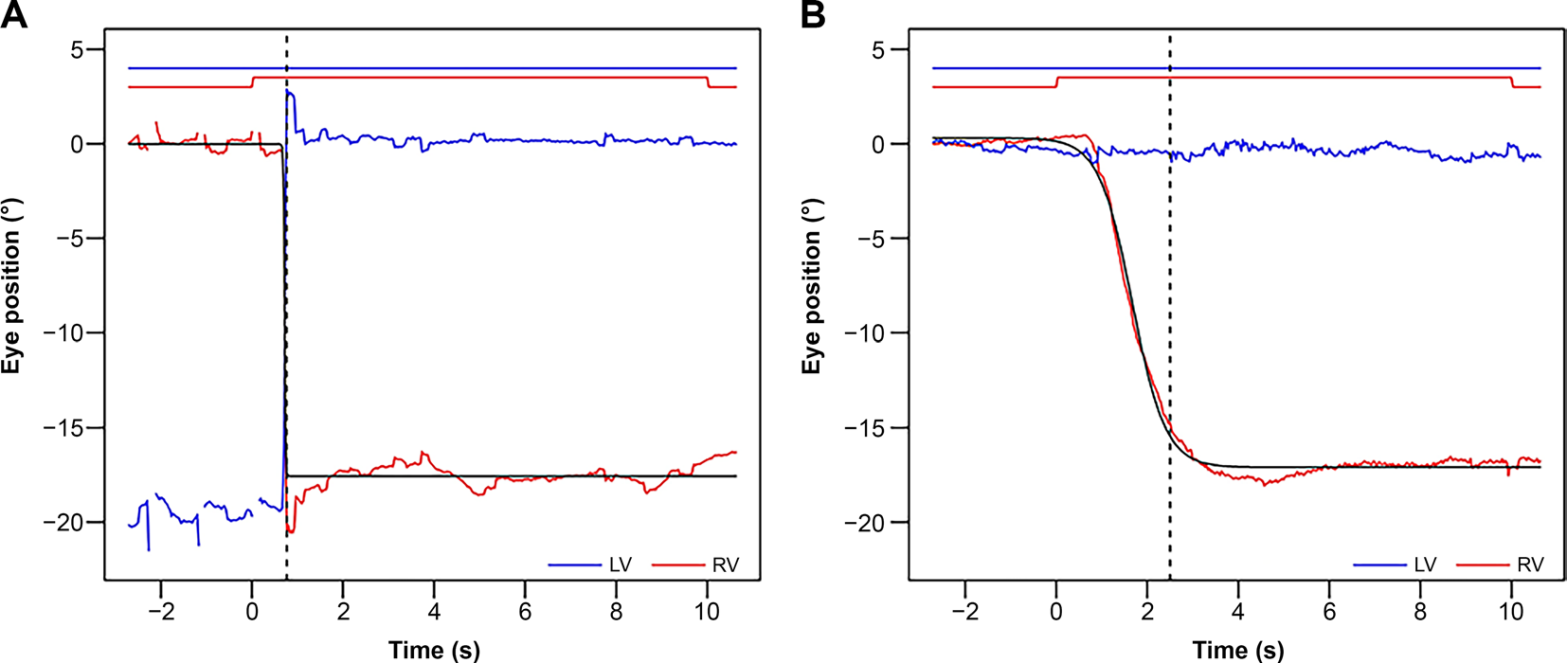
Trajectories of eye deviation in exotropia and intermittent exotropia. Results from the participants with exotropia or intermittent exotropia are shown in the format same to Fig. 1C. As described in Fig. 1C, the straight line at the top of the figure indicates nonocclusion, and the protrusion indicates occlusion. The dotted line indicates the time when the deviation stabilized. Each waveform shows the change in eye position corresponding to the y-axis, and a logistic curve is fitted to the deviation of the right eye. **A**. Results from a participant with exotropia. Prior to occlusion, the right eye was fixated, and immediately after occlusion, the left eye was fixated. **B**. Results from a participant with intermittent exotropia. Prior to occlusion, the participant was in phoria, with both eyes at approximately 0°

All the participants showed eye deviation during monocular occlusion. The data for exotropia (n = 1) is shown in Fig. 2A. In this case, at the binocular eye position (at − 2 to 0 s), the right eye was fixated, while the left eye was deviated. After occluding the right eye (at 0 s), the left eye fixated in turn with a maximum deviation speed of 93.6°/s, showed an overshoot-like waveform (rebound-saccade). The fusion-free eye position stabilized at about 0.8 s (the dotted line in Fig. 2A) with the angle of eye deviation was − 17.4°/s. The data for intermittent exotropia (n = 1) is shown in Fig. 2B. At the binocular eye position, the gaze was stationary at approximately 0° in both eyes, and the eyes were in phoria at the start of occlusion. The maximum deviation speed of 2.7°/s was reached at 1.7 s after the start of occlusion. The fusion-free eye position stabilized at 2.5 s (the dotted line in Fig. 2B), and the angle of eye deviation was − 17.5°/s.

**Fig. 3:**
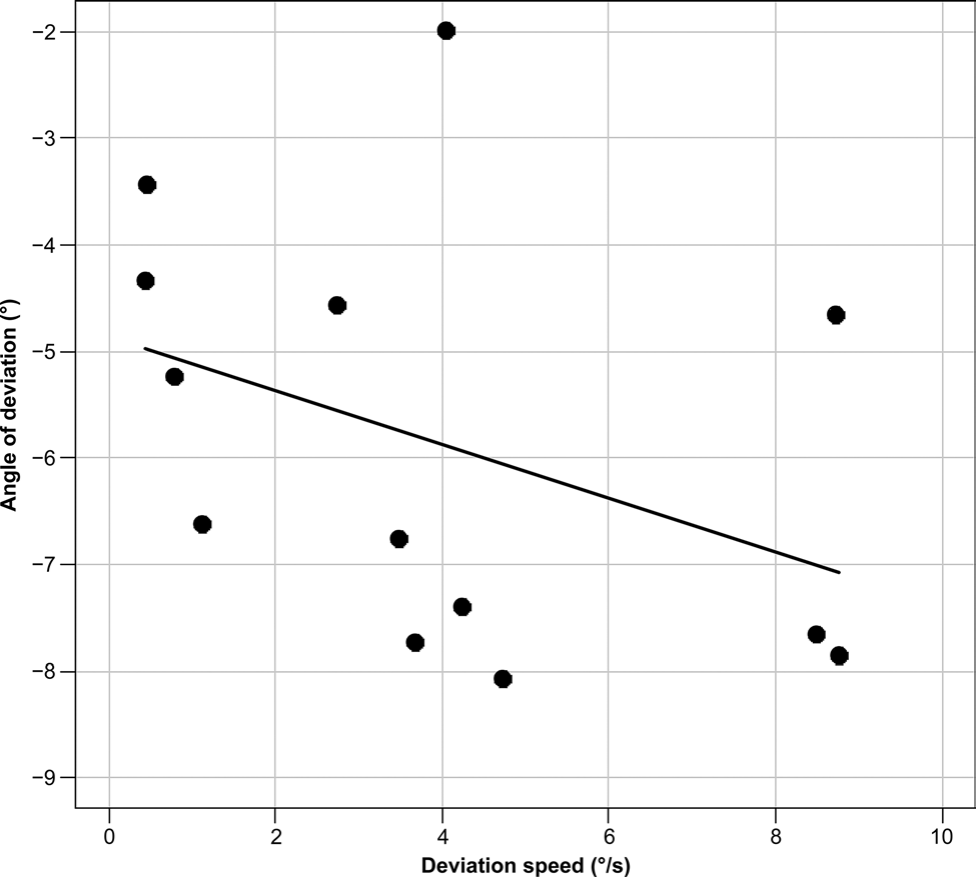
Correlation between the angle of deviation and maximum deviation speed. A significant correlation was seen between the angle of deviation and maximum deviation speed in exophoria as calculated using Spearman’s rank correlation coefficient (r_s_ = − 0.582, *p* = 0.0403)

**Fig. 4:**
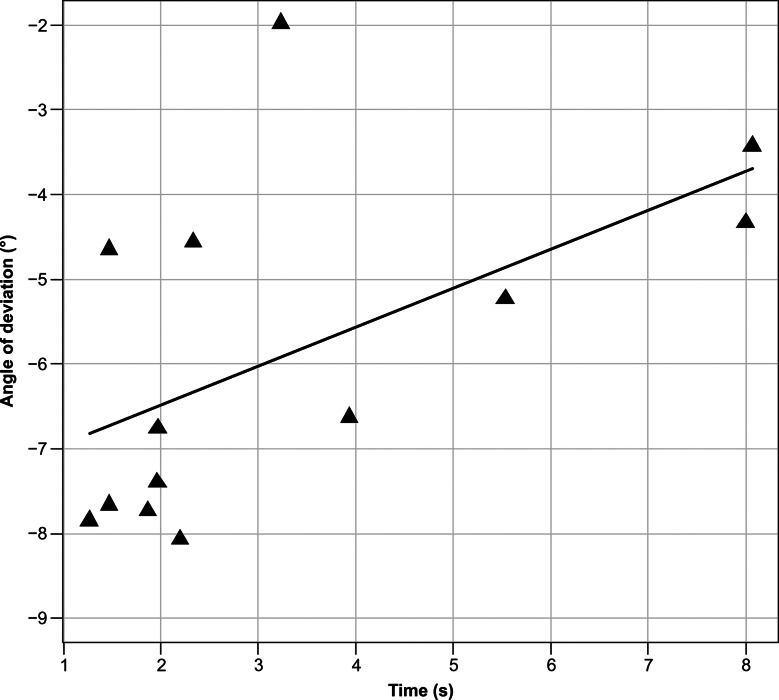
Correlation between the angle of deviation and eye position stabilization time. A significant correlation was seen between the angle of deviation and the time until the deviation stabilized in exophoria as calculated using Spearman’s rank correlation coefficient (r_s_ = 0.663, *p* = 0.0135)

**Fig. 5:**
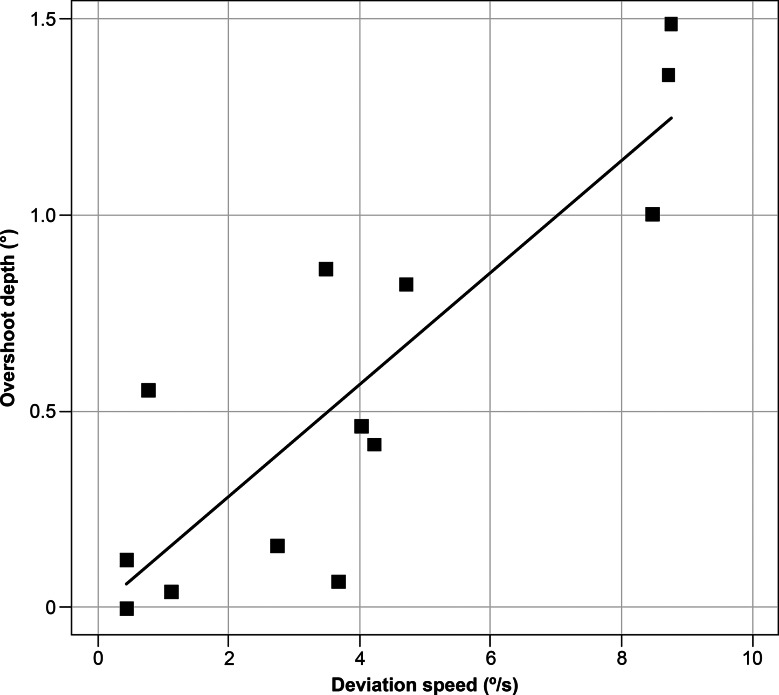
Correlation between overshoot depth and deviation speed. A significant correlation was seen between overshoot depth and deviation speed in exophoria as calculated using Spearman’s rank correlation coefficient (r_s_ = 0.775, *p* = 0.0029)

In total, 13 participants with heterophoria were included. The representative examples shown in Fig. 1C depict a trajectory similar to that of intermittent exotropia, in which the eyes were in phoria at the onset of occlusion. The fusion-free eye position stabilized at 3.33 ± 2.39 s, and the angle of eye deviation was − 5.87 ± 1.96°. The maximum deviation speed was 3.97 ± 3.05°/s. An overshoot of > 1°, 0.5°–1.0°, and < 0.5° was exhibited by 3, 4, and 6 participants, respectively. The overshoot depth was 0.56 ± 0.51°. Significant correlations were found between the angle of eye deviation and deviation speed (r_s_ = − 0.582, *p* = 0.0403) (Fig. 3), between the angle of eye deviation and time until the fusion-free eye position stabilized (r_s_ = 0.663, *p* = 0.0135) (Fig. 4), and between the overshoot depth and deviation speed (r_s_ = 0.775, *p* = 0.0029) (Fig. 5). The APCT results for all participants were − 8.67 ± 10.18 prism diopters (PD), whereas the deviation measured using the ORTe EYENAC was − 7.42 ± 4.47° (− 13.12 ± 8.11 PD). The deviation measured by the ORTe EYENAC was significantly more exotropic than that measured by the APCT (*p* = 0.0004, Paired t-test).

## Discussion

Ocular deviation under occlusion was effectively visualized using the ORTe EYENAC, providing a clear representation of eye movement during the cover test and offering additional information to support the assessment of eye deviation. The prism adaptation test (PAT) and monocular occlusion (MO) are necessary for identifying the maximum ocular deviation angle in patients with intermittent exotropia or exotropia [13]. In this study, a popular eye position test without PAT or MO was performed using an eye-tracking system. The gold standard for quantifying heterotropia and heterophoria is the APCT, which can be performed using only an occluder and prism, but it heavily relies on the skill of the examiner. The results of APCT performed by 2 experienced strabismus surgeons in patients with abducens nerve palsy showed that the 95% limits of agreement (LOA) half-widths were 10.2 PD at distance and 9.2 PD at near [10]. The Pediatric Eye Disease Investigator Group [2] reported that the 95% LOA for a difference between 2 APCT measurements among children with esotropia were ± 10.4 PD at distance and ± 11.7 PD at near for angles greater than 20 PD, while these were ± 5.8 PD at distance and ± 4.7 PD at near for angles between 10 and 20 PD. Since the results of the APCT can vary, inaccuracies can lead to poor strabismus surgery outcomes and inappropriate prism lenses. Therefore, more objective methods using modern technology are needed to quantify heterotopia and heterophoria.

VOG technology has evolved significantly over the past decade and is now widely used in eye position testing. Park et al. [14] reported that the calculated values obtained from VOG tended to be similar to those obtained using APCT. Moreover, Cantó-Cerdán et al. [15] reported that measuring strabismus between VOG and APCT for esotropia and exotropia yielded an excellent correlation and good agreement between both methods, especially for exotropia. In our study, eye position was quantified using a method for eye-tracking under occlusion during CUT.

In the cover test, fusion is eliminated by occluding one eye, allowing sufficient ocular deviation to be detected. However, in measuring fusion-free eye position, there is no standard occlusion time set for each eye. Holmes et al. [10] suggested that the results of the APCT may be affected based on the period of dissociation, possibly due to an underlying phoric component. Such variabilities in measurement technique may lead to differences in eye deviation measured on the APCT. Thus, to eliminate interrater variability during the periods of dissociation and accurately measure the fusion-free eye position, it is necessary to clarify the time required for the eye position to stabilize under occlusion. However, since the cover test cannot observe eye deviation under occlusion, the time it takes for eye deviation to stabilize after the start of occlusion is unknown. In this study, the fusion-free eye position stabilized at about 0.8 s in exotropia and 3.33 ± 2.39 s in exophoria. In the exophoria group, the smaller the angle of eye deviation, the slower the deviation speed and the longer it took to stabilize the deviation. Therefore, the occlusion time required for the angle of deviation to stabilize depends on the amount of deviation.

A comparison between the deviations measured by the APCT and the ORTe EYENAC revealed that the latter produced significantly more exotropic results. Iwata et al. [11] previously reported that measurements obtained with a Hess screen test and an objective nine-gaze-direction measuring device (OMD; the prototype of the ORTe EYENAC) showed more exotropic deviations with the OMD. They suggested that this difference was likely due to the Hess screen test being performed at a distance of 1.4 m with a screen in front of the subject, while the OMD displayed stimuli directly in front of each eye, resulting in less convergence influence. Similarly, in the present study, the APCT was performed using a near target (33 cm), which may have induced convergence, potentially explaining the difference in results.

During the prism cover test in patients with exotropia, the eye deviates from outward to inward. However, this can be excessive in some patients, leading to a corrective saccade from inward to outward, termed by Mehdorn as a rebound-saccade [16]. In our study, the participants with exotropia also changed fixation by saccades at a maximum deviation speed of 93.6 deg/s at the same time the occlusion began, and after showing a rebound-saccade, the eye position stabilized. Moreover, some participants showed an overshoot-like waveform in exophoria. However, the overshoot-like waveform in the exophoria was different from the rebound-saccade in exotropia and had a different waveform from the movement of a corrective saccade. Notably, overshoot depth was significantly correlated with faster eye deviation speed in those with exophoria. A previous experiment investigated changes in the eyeball after pulling it outwards while anesthetized. This revealed that the speed of displacement increased as the pulling force became stronger, but no overshoot-like waveform was observed [17]. This suggests that the overshoot-like waveform in exophoria cannot be attributed to the mechanical properties of the eye. A convergence factor is likely involved, but further investigation is needed to confirm this since the current system cannot record the pupil diameter over time. In the future, once the device can record changes in pupil diameter over time, the possible involvement of the near response center can be confirmed.

The limitation of this study was its small sample size, which included only 13 subjects. While significant correlations make a type 1 error highly unlikely, a larger sample size would provide more robust results and improve the generalizability of the findings. Additionally, the results are applicable only to participants with exodeviation, limiting their ability to generalize to other eye conditions. Future studies should examine the trajectory of eye deviation in other types of positional abnormalities, including heterotropia and other forms of strabismus. Moreover, we are working on improving the system to incorporate recordings of pupil responses and Electroencephalogram data, which could offer more comprehensive insights into eye movements and related parameters.

## Conclusions

By using the ORTe EYENAC, it was possible to observe ocular deviation under occlusion of one eye, which cannot be observed with CUT, and the amount of deviation could be quantified. Among participants with exophoria, the fusion-free eye position stabilized at 3.33 ± 2.39 s after occlusion. However, since the stabilization time correlated with the angle of deviation, which also correlated with deviation speed, the required time for occlusion depends on the magnitude of the participant’s angle of deviation.

## Data Availability

The datasets used and analyzed during the current study are available from the corresponding author upon reasonable request.

## Abbreviations

CUT: Cover–uncover test
APCT: Alternate prism cover test
SPCT: Single prism cover test
VOG: Video-oculography
LCD: Liquid crystal display
PD: Prism diopters
PAT: prism adaptation test
MO: Monocular occlusion
LOA: Limits of agreement
